# Methyl Gallate Inhibits Osteoclast Formation and Function by Suppressing Akt and Btk-PLCγ2-Ca^2+^ Signaling and Prevents Lipopolysaccharide-Induced Bone Loss

**DOI:** 10.3390/ijms18030581

**Published:** 2017-03-07

**Authors:** Jong Min Baek, Ju-Young Kim, Chang Hoon Lee, Kwon-Ha Yoon, Myeung Su Lee

**Affiliations:** 1 Department of Anatomy, School of Medicine, Wonkwang University, Iksan, Jeonbuk 570-749, Korea; phone8418@hanmail.net; 2 Imaging Science-based Lung and Bone Diseases Research Center, Wonkwang University, Iksan, Jeonbuk 570-749, Korea; kimjy1014@gmail.com (J.-Y.K.); lch110@wku.ac.kr (C.H.L.); khy1646@wku.ac.kr (K.-H.Y.); 3 Department of Radiology, School of Medicine, Wonkwang University, Iksan, Jeonbuk 570-749, Korea; 4 Division of Rheumatology, Department of Internal Medicine, Wonkwang University, Iksan, Jeonbuk 570-749, Korea

**Keywords:** methyl gallate, osteoclast, Akt, Ca^2+^ signaling, bone resorption, osteoporosis

## Abstract

In the field of bone research, various natural derivatives have emerged as candidates for osteoporosis treatment by targeting abnormally elevated osteoclastic activity. Methyl gallate, a plant-derived phenolic compound, is known to have numerous pharmacological effects against inflammation, oxidation, and cancer. Our purpose was to explore the relation between methyl gallate and bone metabolism. Herein, we performed screening using methyl gallate by tartrate resistant acid phosphatase (TRAP) staining and revealed intracellular mechanisms responsible for methyl gallate-mediated regulation of osteoclastogenesis by Western blotting and quantitative reverse transcription polymerase chain reaction (RT-PCR). Furthermore, we assessed the effects of methyl gallate on the characteristics of mature osteoclasts. We found that methyl gallate significantly suppressed osteoclast formation through Akt and Btk-PLCγ2-Ca^2+^ signaling. The blockade of these pathways was confirmed through transduction of cells with a CA-Akt retrovirus and evaluation of Ca^2+^ influx intensity (staining with Fluo-3/AM). Indeed, methyl gallate downregulated the formation of actin ring-positive osteoclasts and resorption pit areas. In agreement with in vitro results, we found that administration of methyl gallate restored osteoporotic phenotype stimulated by acute systemic injection of lipopolysaccharide in vivo according to micro-computed tomography and histological analysis. Our data strongly indicate that methyl gallate may be useful for the development of a plant-based antiosteoporotic agent.

## 1. Introduction

The development of rigidity and healthy conditions in the skeletal system are mainly influenced by a continuous and complex process, bone remodeling, which is dependent on systemic energy metabolism [1]. The bone remodeling cycle is a series of consecutive phases and is composed of activation, resorption, reversal, and formation. The activation phase is regulated by the effects of local transforming growth factor beta1 (TGF-β1) on mesenchymal stem cells of the osteoblast lineage. These cells interact with hematopoietic precursor cells to generate osteoclasts in the resorption phase. Subsequently, osteoclasts derived from mononuclear stromal cells are attached to the surface of the bone matrix in the reversal phase and complete the resorption stage, which is followed by the production of initial signals for differentiation of mesenchymal stem cells into osteoblasts. Eventually, functional osteoblasts lie at the surface of the existing matrix and then accumulate fresh layers of bone in the formation phase. This tight orchestration of each phase essentially requires the balance between osteoclast-mediated bone-resorptive activity and osteoblast-mediated bone formative activity [2,3,4].

Differentiation of macrophage precursors into multinucleated active osteoclasts initially proceeds through the stimulation of committed stromal-osteoblast lineage cells [5]. A membrane-bound form of a cytokine, receptor activator of nuclear factor κB (RANK), which is expressed in osteoclastic precursors, binds to its partner, RANK ligand (RANKL) derived from the surface of osteoblastic precursors, resulting in differentiation into (and activation of) osteoclasts. Meanwhile, osteoblasts also release a soluble decoy receptor of RANKL, osteoprotegerin, which acts as an antagonist of the interaction between RANK and RANKL. Under the tight control of these essential cytokines, osteoclast progenitors with macrophages can differentiate into mature osteoclasts that digest the bone matrix through secretion of protons into the extracellular compartment [6,7,8,9].

In the case that the bone-resorbing activity is excessive with abnormal development, there is a risk of osteoporosis leading to the loss of bone density, deterioration of bone microarchitecture, and consequent fragility fracture [10,11]. At present, there are two types of medications that are currently used in the treatment of osteoporosis. One is antiresorptive agents that decrease the ratio of bone loss by suppressing osteoclastic bone resorption, and the other is anabolic agents that increase the ratio of bone formation by enhancing density, connectivity, and geometric features in the microstructure of bone [12,13]. Nevertheless, there are severe adverse effects such as an increased risk of serious infections in some patients prescribed denosumab, or hypercalcemia with acute renal failure in the medication group of parathyroid hormone for osteoporosis [14,15]. Hence, to counteract these side effects, numerous researchers have tried to develop novel therapeutic agents for bone metabolic disorders such as osteoporosis on the basis of natural plant-derived compounds [16,17,18].

For the purpose of proposing a promising candidate compound for osteoporosis, in this study, we performed experiments on methyl gallate. The latter is a phytochemical and polyphenolic compound that is widely believed to have medicinal properties such as antioxidant [19], antitumor [20], anti-inflammatory [21], and antimicrobial [22]. In addition, it has been proven that methyl gallate acts as a potent inhibitor of sodium and potassium channels in skeletal muscle cells [23]. There have been no studies on the relation between methyl gallate and osteoclasts. Hence, this study was designed to verify the effect of methyl gallate not only on RANKL-mediated differentiation of bone marrow macrophages (BMMs) into osteoclasts and its intracellular mechanisms in vitro but also on lipopolysaccharide (LPS)-induced bone loss in a mouse model (in vivo) to determine the possible therapeutic value of methyl gallate as a novel safe treatment of osteoporosis.

## 2. Results

### 2.1. Methyl Gallate Attenuates Receptor Activator of Nuclear Factor KappaB Ligand (RANKL)-Mediated Osteoclastogenesis by Suppressing Both mRNA and Protein Expression of c-Fos and Nuclear Factor of Activated T-Cells c1(NFATc1)

**Figure 1:**
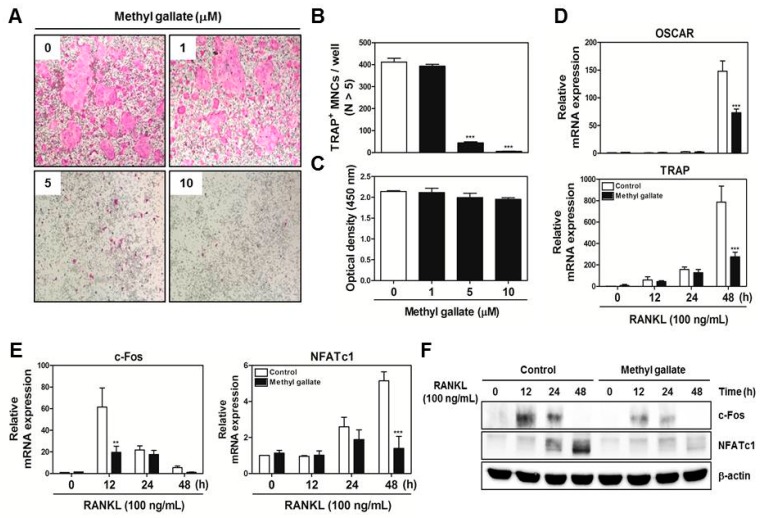
Methyl gallate inhibits receptor activator of nuclear factor κB ligand (RANKL)-induced osteoclastic differentiation through downregulation of c-Fos and NFATc1. (**A**) bone marrow macrophages (BMM)s were cultured with M-CSF (30 ng/mL) and RANKL (100 ng/mL) in the presence or absence of the indicated concentrations of methyl gallate. The cells were fixed, permeabilized, and stained with a tartrate resistant acid phosphatase (TRAP) solution. TRAP-positive multinucleated cells (MNCs) were photographed under a light microscope at the indicated magnification (10×); (**B**) The number of TRAP-positive MNCs (nuclei >5) was determined in these cultures. *** *p* < 0.001 vs. DMSO-treated control group; (**C**) BMMs were seeded into 96-well plates and cultured for 3 days in the presence of M-CSF (30 ng/mL) with the indicated concentrations of methyl gallate. After that, cell viability was analyzed by an tetrazolium salt sodium 3′-{1-[(phenylamino)-carbonyl]-3,4-tetrazolium}-*bis* (4-methoxy-6-nitro)benzene-sulfonic acid hydrate (XTT) assay; (**D**) BMMs were preincubated with or without methyl gallate (10 µM) for 1 h in the presence of M-CSF (30 ng/mL) and then stimulated with RANKL (100 ng/mL) for the indicated periods. The mRNA expression levels of osteoclast-associated receptor (OSCAR) and TRAP were analyzed by quantitative real-time RT-PCR. *** *p* < 0.001 vs. the control group at the corresponding time point; (**E**) The mRNA expression levels of c-Fos and NFATc1 were analyzed by quantitative real-time RT-PCR. ** *p* < 0.01; *** *p* < 0.001 vs. control group at the corresponding time point; (**F**) BMMs were preincubated with or without methyl gallate (10 µM) for 1 h in the presence of M-CSF (30 ng/mL) before RANKL (100 ng/mL) stimulation at the indicated time points. Whole-cell lysates were analyzed by Western blotting with the indicated antibodies. β-Actin served as internal control.

To examine the effect of methyl gallate on RANKL-induced osteoclast differentiation, various concentrations (0, 1, 5, and 10 µM) of methyl gallate were incubated with BMMs in the presence of M-CSF (30 ng/mL) and RANKL (100 ng/mL). The results showed that methyl gallate reduced formation of osteoclast-like cells in a dose-dependent manner (Figure 1A). 

The number of tartrate resistant acid phosphatase (TRAP)-positive multinucleated cells (MNCs) was significantly decreased in BMM cultures treated with methyl gallate at the concentrations of 5 and 10 µM (Figure 1B). This inhibitory action of methyl gallate did not have any cytotoxic effect (Figure 1C). Furthermore, the quantified confirmation of the involvement of methyl gallate in osteoclastic differentiation was tested by quantitative RT-PCR assay. The results proved that methyl gallate inhibited mRNA expression of osteoclast-associated receptor (OSCAR) and TRAP, which are specific for authentic osteoclasts at the transcriptional level (Figure 1D). It is well established that expression of osteoclast marker genes is mainly regulated by NFATc1. Hence, we performed quantitative RT-PCR and Western blotting to reveal the possibility that methyl gallate is linked with master regulators of osteoclastogenesis: c-Fos and NFATc1. As shown in Figure 1E,F, methyl gallate diminished both mRNA and protein expression levels of these two specific transcription factors as expected. These phenomena revealed that methyl gallate blocked the RANKL-dependent induction of the transcription factors and subsequent osteoclast marker genes, leading to an inhibitory effect on osteoclast formation.

### 2.2. Methyl Gallate Decreases Phosphorylation of Akt, Btk, and PLCγ2 in RANKL-Induced Signaling Transduction Pathways

**Figure 2:**
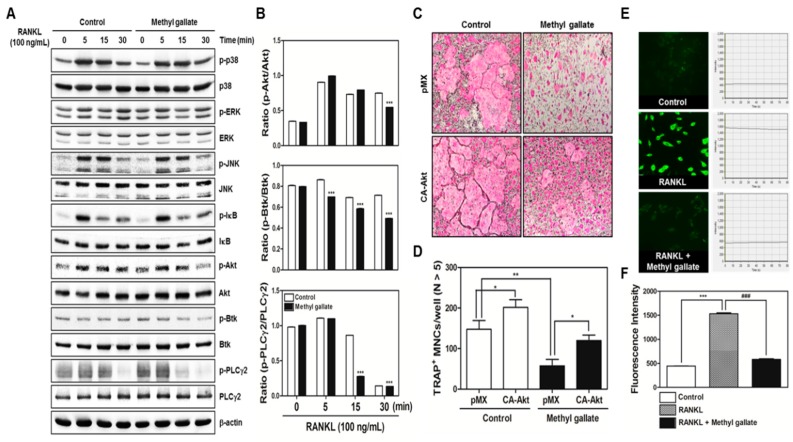
Methyl gallate suppresses phosphorylation of Akt, Btk, and PLCγ2. (**A**) BMMs were preincubated with or without methyl gallate (10 µM) for 1 h in the presence of M-CSF (30 ng/mL) before RANKL (100 ng/mL) stimulation at the indicated time points. Whole-cell lysates were analyzed by Western blotting with the indicated antibodies. β-Actin was used as internal control; (**B**) Quantification of relative ratio of band intensity was performed using Image J software. *** *p* < 0.001; (**C**) BMMs were infected with retroviruses expressing pMX-IRES-EGFP (pMX) and pMX-CA-Akt-EGFP. The infected BMMs were cultured with or without methyl gallate (10 µM) in the presence of M-CSF (30 ng/mL) and RANKL (100 ng/mL) for 4 days. After that, the cells were fixed and stained with a TRAP solution. TRAP-positive cells were photographed under a light microscope at the indicated magnification (10×); (**D**) The number of TRAP-positive MNCs (nuclei > 5) was counted in these cultures. * *p* < 0.05; ** *p* < 0.01; (**E**) BMMs were incubated with M-CSF (30 ng/mL) and RANKL (100 ng/mL) in the presence or absence of methyl gallate (10 µM) for 3 days. For Ca^2+^ quantification, the cells were incubated with Fluo-3/AM for 30 min in serum-free-MEM followed by confocal imaging analysis at the indicated magnification (40×). Each line shows the fluorescence intensity of the respective condition. The fluorescence intensity in each group was recorded within 80 s; (**F**) The intensity of Fluo-3/AM was statistically analyzed at time point 80 s. *** *p* < 0.001; ^###^ *p* < 0.001.

In the next phase, to elucidate the molecular mechanism underlying the methyl gallate-mediated early stage of osteoclastogenesis, we tested the effect of methyl gallate on phosphorylation of early cellular transducers, including mitogen-activated protein (MAP) kinases (including p38, ERK, and JNK), and IκB, Akt, as well as Btk and PLCγ2. As shown in Figure 2A,B, increased phosphorylation of Akt and Ca^2+^-dependent signaling molecules such as Btk and PLCγ2 mediated by RANKL stimulation in the control group was blocked by methyl gallate. In accordance with this phenomenon, we conducted additional experiments to confirm the effects of methyl gallate on the early signaling pathways in osteoclastic differentiation. By applying a retroviral transfection-based assay to induce overexpression of Akt in BMMs, we found that ectopic expression of Akt was sufficient to reverse the suppressive effect of methyl gallate on the formation of TRAP-positive osteoclasts (Figure 2C,D). Moreover, owing to the relation between methyl gallate and Ca^2+^ signaling uncovered by Western blotting, we then tested whether intracellular Ca^2+^ influx was regulated by methyl gallate. As shown in Figure 2E, the control group treated with only M-CSF (negative control) did not display Ca^2+^ influx owing to the absence of RANKL that leads to significant elevation of intracellular Ca^2+^ influx, while the RANKL group treated with both M-CSF and RANKL (positive control) showed an increased level of intracellular Ca^2+^ stained with Fluo-3/AM. Compared with the RANKL group, methyl gallate markedly suppressed the total amount of Ca^2+^ entering the preosteoclasts. Additionally, we recorded the intensity of green fluorescence of five individual cells in each group within 80 s and built a graph that represents statistical quantification at the time point of 80 s (Figure 2F). These results clearly showed that methyl gallate attenuated RANKL-induced osteoclastogenesis by inhibiting both Akt phosphorylation and intracellular Ca^2+^ influx mediated by Btk and PLCγ2.

### 2.3. Methyl Gallate Restrains F-Actin Ring Structure and Bone-Resorbing Activity of Mature Osteoclasts by Decreasing mRNA Expression of Osteoclast-Specific Gene Markers

**Figure 3:**
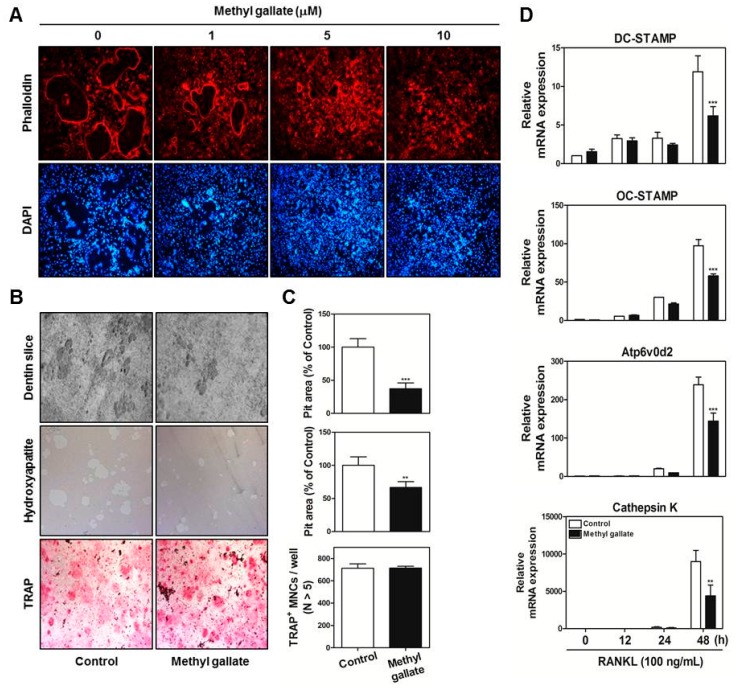
Methyl gallate attenuates F-actin ring formation and bone-resorbing activity of mature osteoclasts. (**A**) BMMs were cultured with M-CSF (30 ng/mL) and RANKL (100 ng/mL) in the presence of the indicated concentrations of methyl gallate. The cells were fixed, permeabilized, and stained with phalloidin and 4′,6-diamidino-2-phenylindole (DAPI). The cells were examined under a confocal laser scanning microscope at the indicated magnification (10×); (**B**) Mature osteoclasts from the coculture system were seeded in 48-well plates with incubation for 6 h, in hydroxyapatite-coated plates with incubation for 24 h, or on dentin slices with incubation for 48 h with or without methyl gallate (10 µM). After that, cells attached to 48-well plates were stained with a TRAP solution, and the cells on hydroxyapatite-coated plates or dentin slices were removed and photographed under a light microscope at the indicated magnification (10×); (**C**) Pit areas on hydroxyapatite-coated plates or dentin slices were quantified using the Image Pro-PLUS (ver. 4.5) software, and the number of TRAP-positive NMCs (nuclei > 5) was determined. *** *p* < 0.001 vs. control group; (**D**) The mRNA expression levels of DC-, OC-STAMP, Atp6v0d2, and Cathepsin K were analyzed by quantitative real-time RT-PCR. ** *p* < 0.01; *** *p* < 0.001 vs. control group at the corresponding time point.

Next, we determined whether methyl gallate interferes with the most characteristic features of mature osteoclasts: the ring-like F-actin structure and bone resorption. As shown in Figure 3A, formation of the F-actin structure by fusion of osteoclasts was suppressed by methyl gallate in a dose-dependent manner. Moreover, we seeded mature osteoclasts derived from the coculture system on dentin slices, hydroxyapatite-coated plates, and 48-well plates in the presence or absence of 10 µM methyl gallate to reveal a possible direct effect of methyl gallate on bone-resorbing activity and survival of mature osteoclasts.

The results suggested that considerable formation of resorption pit areas was taking place in the control group of dentin slices and hydroxyapatite-coated plates; however, methyl gallate significantly disrupted the ability of mature osteoclasts to form resorption pits in these cultures. Through TRAP staining analysis, we confirmed that the antiresorptive action of methyl gallate did not exert any cytotoxicity on mature osteoclasts (Figure 3B,C). In line with these phenomena, the quantitative RT-PCR data proved that methyl gallate decreased the mRNA expression of several transcription factors associated with cell–cell fusion and osteoclastic bone resorption: DC-, OC-STAMP, Atp6v0d2, and Cathepsin K (Figure 3D). This finding clearly showed that methyl gallate attenuated expression of DC-, OC-STAMP, Atp6v0d2, and Cathepsin K, negatively influencing not only the formation of F-actin ring-positive osteoclasts but also their ability to dissolve bone.

### 2.4. Administration of Methyl Gallate Reverses Lipopolysaccharide (LPS)-Mediated Inflammatory Bone Erosion In Vivo

**Figure 4:**
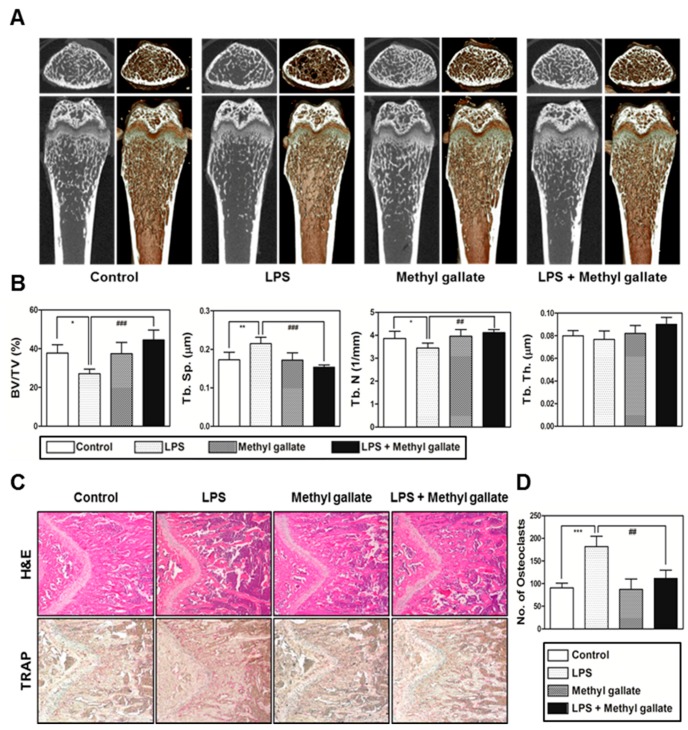
Methyl gallate restores the lipopolysaccharide (LPS)-mediated bone erosion mice model. (**A**) Mice were euthanized on day 8 after first LPS injection and 2D or 3D radiographs of the coronal and transverse sections of the proximal femurs were obtained with a micro-CT scanner; (**B**) The BV/TV, Tb.Sp, Tb.Th, and Tb.N of the femurs were determined by analyzing the micro-CT data in the INFINITT-Xelis software. * *p* < 0.05; ** *p* < 0.01; ^##^ *p* < 0.01; ^###^ *p* < 0.001; (**C**) Dissected femurs were fixed, decalcified, embedded, and sectioned. The slices were stained with TRAP (bottom) and hematoxylin & eosin (H&E) (top), and photographed under a light microscope at the indicated magnification (10×); (**D**) The number of osteoclasts per visual field of tissues was measured by histomorphometric analysis. *** *p* < 0.01; ^##^ *p* < 0.01.

To determine whether the in vitro effect of methyl gallate on osteoclastic differentiation and function can be true in vivo, we used mice with LPS-induced bone loss as a model of osteoporosis based on induced inflammation. The mice were injected with LPS intraperitoneally and were orally treated with methyl gallate or phosphate buffered saline (PBS). After 8 days, the left femurs of euthanized mice were analyzed using a micro-computed tomography (CT) system and the right femurs were stained with TRAP and hematoxylin and eosin (H&E). Although the treatment with LPS reduced bone mass in femurs of mice compared with the control group, 2D and 3D visualization indicated that there was a partial recovery of bone volume in the LPS-and-methyl gallate-treated group of mice (Figure 4A). 

Morphometric analysis of the femurs of the LPS-treated group of mice showed a decreased level of BV/TV and Tb.N as well as an increased level of Tb.Sp. In contrast, significant restoration of BV/TV, Tb.N, and Tb.Sp was detected in the LPS group treated with methyl gallate (Figure 4B). Furthermore, histological analysis confirmed that treatment with methyl gallate reversed the LPS-mediated loss of the trabecular bone matrix within growth plates and suppressed TRAP-positive osteoclast formation in vivo (Figure 4C). As we anticipated, there was an obvious increase in the number of osteoclasts per visual field in the LPS-treated group in comparison with the control, and this increasing trend was effectively blocked by methyl gallate (Figure 4D). As a result, in accordance with our in vitro experiments, it was demonstrated that methyl gallate has an inhibitory effect on osteoclast formation and subsequent bone-resorbing activity in vivo.

## 3. Discussion

In the present study, we confirmed that methyl gallate suppressed activation of two distinct pathways including Akt and Ca^2+^ signaling that are mediated by Btk and PLCγ2 in primary murine BMMs during osteoclastogenesis. In agreement with the results, the overexpression of Akt induced by retroviral transduction was apparently sufficient to reverse the antiosteoclastic effects of methyl gallate. Besides, the fluorescence intensity of intracellular Ca^2+^ influx stained with Fluo-3/AM was effectively decreased by treatment with methyl gallate. Owing to this defective signal transduction in the early phase of osteoclastic differentiation, the c-Fos-NFATc1 transcriptional activation cascade was blocked, disrupting the subsequent expression of various transcription factors driving osteoclastic differentiation and function, for example, OSCAR, TRAP, DC-, OC-STAMP, Atp6v0d2, and Cathepsin K. Additionally, we found that methyl gallate is a potent inhibitor of the organization of F-actin ring cytoskeleton and osteoclastic bone-resorbing activity. According to the antiosteoclastic properties of methyl gallate in vitro, we next evaluated the potential antiosteoporotic action of methyl gallate, using a mouse model of inflammation-mediated osteoporosis. Our results showed that methyl gallate altered pathological conditions of the bone microenvironment induced by systemic injection of LPS in vivo. To the best of our knowledge, the present study for the first time shows that methyl gallate has a strong effect on bone metabolism and pathogenesis of skeletal disorders such as osteoporosis.

There are two main steps in the process of osteoclastic differentiation. One is the regulation of various transcriptional activities at the early stages. The other is the cellular fusion of osteoclasts and macrophage giant cells to form multinucleated giant bone-digesting cells at the late stages [24]. To initiate the commitment and differentiation of osteoclast-like cells, the binding of RANKL derived from osteoblast-like cells and its partner receptor, RANK located in the membrane of osteoclast precursor cells, is primarily required, leading to the induction of various early signaling cascades [5,6]. From our results, we found out that methyl gallate inhibits RANKL-induced phosphorylation of Akt (Figure 2). Among the RANKL-dependent signal transducers, Akt (also known as protein kinase B; PKB) in BMMs is known to play a critical role in osteoclastic differentiation and survival according to the literature. It was reported that Akt deficiency abrogates differentiation into osteoclasts by controlling nuclear localization of NFATc1 in response to decreased expression of RANKL in vitro; a pathological phenotype including impaired bone development and dwarfism is observed in mice lacking Akt in vivo [25,26,27]. Furthermore, the clinical importance of Akt was demonstrated by elucidating the therapeutic action of systemic administration of Akt inhibitor LY294002 on multiple myeloma (MM)-mediated osteoclast formation in severe combined immunodeficiency (SCID) mice [28]. Besides Akt signaling, methyl gallate also attenuated phosphorylation of Btk and PLCγ2, decreasing intracellular Ca^2+^ concentration (Figure 2). Ca^2+^ signaling is one of the key downstream processes in the RANKL/RANK pathway, playing a pivotal role in osteoclastic differentiation and functions [29]. During RANKL-mediated differentiation into osteoclasts, phosphorylation of PLCγ2 is induced in response to the activation of Btk and Tec tyrosine kinases or transactivation of immunoreceptor tyrosine-based activation motif (ITAM)-associated receptors such as costimulatory receptors, (e.g., triggering receptor expressed on myeloid cells 2; TREM2) and subsequently, a release of Ca^2+^ is evoked, enhancing the magnitude of the Ca^2+^ influx. Upon activation of this process, mobilization of cytosolic Ca^2+^ is triggered, and this oscillation gives rise to prolonged signaling of Ca^2+^, bringing about increased expression of NFATc1 [29,30,31,32]. It was described elsewhere that the blockade of the Ca^2+^-release-activated Ca^2+^ (CRAC) channel (which is one of the main ways of Ca^2+^ entry) inhibited the formation of multinucleated osteoclasts [33]. Herein, we confirmed the attenuation of Akt phosphorylation and Ca^2+^ influx via Btk-PLCγ2-Ca^2+^ signaling, resulting in downregulation of NFATc1 as molecular mechanisms underlying the inhibitory effect of methyl gallate on RANKL-induced osteoclastogenesis (Figure 1 and Figure 2). Although we did not elucidate the direct target of methyl gallate in osteoclast differentiation, we proposed the possible mechanisms underlying methyl gallate-mediated inhibition of RANKL-dependent osteoclastogenesis. In intracellular signaling pathways in osteoclasts, c-Src kinase is well known to induce not only Akt phosphorylation through phosphoinositide-3 kinase (PI3K), but Btk-PLCγ2 signaling via integrin αv/β3-induced Syk phosphorylation [34,35]. Hence, we assumed that the direct interaction of methyl gallate with c-Src or targeting both Akt and Syk was what triggered the methyl gallate-mediated Akt and Btk-PLCγ2-Ca^2+^ signaling.

At the late stages of osteoclastogenesis, actin cytoskeletal reorganization (which is closely related to mammalian cell activities including movement, fusion, and adhesion) is required. To reorganize the stable actin structure, regulation of actin filament dynamics is a crucial stage through the coordination of the activities of different types of actin-binding proteins [36,37,38]. After rearrangements of the F-actin ring cytoskeleton (which are a prerequisite for osteoclast-mediated bone digestion), formation of the sealing zone and ruffled border is required for adhesion to the extracellular bone matrix [39,40]. The actin-rich sealing zone generated by osteoclasts is composed of high-density packed podosome-like units and is intimately associated with osteoclast attachment to the bone surface by secreting cell surface receptors such as members of the integrin family. In addition, a ruffled border (which shows features of a late endosomal/lysosomal bone facing membrane domain) is located in the center of the sealing zone for degradation of the bone matrix by secreting proton ions and proteinase [39,40,41,42]. In the present study, our results provide evidence that methyl gallate suppresses (1) the formation of F-actin ring-positive osteoclasts stained with phalloidin in a dose-dependent manner; and (2) the bone-digestive ability of mature osteoclasts from the coculture system of osteoclast precursors/osteoblasts (Figure 3). 

In summary, our study clearly showed that methyl gallate attenuates RANKL-dependent osteoclastic differentiation via Akt and Btk-PLCγ2-Ca^2+^ signaling and characteristics of osteoclast maturation including F-actin structure and bone-resorbing activity in vitro. Reflecting these phenomena, administration of methyl gallate effectively attenuated the LPS-induced osteoporotic phenotype in vivo. Although, in this study, we focused on the therapeutic value of methyl gallate on inflammatory osteoporosis, our further study needs to investigate the effect of methyl gallate on other kinds of osteoporosis models, such as ovariectomized (OVX) mice. Collectively, these findings suggest that methyl gallate is one of the candidates for plant-based therapeutics for skeletal disorders such as osteoporosis. 

## 4. Materials and Methods

### 4.1. Reagents and Antibodies

Methyl gallate (98% purity) was purchased from Sigma-Aldrich (St. Louis, MO, USA) and dissolved in dimethyl sulfoxide (DMSO). Recombinant soluble human macrophage colony-stimulating factor (M-CSF) and RANKL were obtained from PeproTech EC Ltd. (London, UK). A monoclonal antibody against β-actin was obtained from Sigma-Aldrich (St. Louis, MO, USA). Antibodies against phosphorylated (phospho)-p38, p38, phospho-extracellular signal-regulated protein kinase (ERK) 1/2, ERK 1/2, phospho-JNK, JNK, phospho-IκB, phospho-Akt, Akt, and Bruton’s tyrosine kinase (Btk) were purchased from Cell Signaling Technology Inc. (Beverly, MA, USA). Antibodies against c-Fos, nuclear factor of activated T cells c1 (NFATc1), IκB, phospho-PLCγ2, and PLCγ2 were purchased from Santa Cruz Biotechnology (Santa Cruz, CA, USA). An antibody against phospho-Btk was purchased from GeneTex (Irvine, CA, USA). Two kinds of secondary antibodies including horseradish peroxidase–conjugated sheep anti-mouse IgG antibody and donkey anti-rabbit immunoglobulin antibody were obtained from Enzo Life Sciences (Farmingdale, NY, USA). Fetal bovine serum (FBS), α-minimum essential medium (α-MEM), penicillin, and streptomycin were purchased from Gibco BRL (Grand Island, NY, USA). All other chemicals were of analytical grade or complied with the standards required for cell culture experiments.

### 4.2. Ethics Statement

Experimental procedures were approved by the Institutional Animal Care and Use Committee (IACUC) of Wonkwang University (Permit number: WKU-16-91). The surgical procedure was performed under sodium pentobarbital anesthesia, and every effort was made to minimize the suffering of the animals. The mice were monitored daily to check their health status.

### 4.3. Experimental Animals 

The mice used in this experiment were 5-week-old male mice of the imprinting control region (ICR) strain weighing 30 ± 2 g that were purchased from Samtako Co., Ltd. (Osan, Korea). During the study period, the mice were housed under conditions of controlled temperature (22–24 °C) and humidity (55%–60%) in a 12:12 h light/dark cycle in small shoe box cages with a layer of sawdust and given free access to sterilized water and standard rodent chow (Samtako Co., Ltd., Osan, Korea). In each cage, five mice were housed and given the opportunity to build a nest for sleeping and thermoregulation; we replaced contaminated sawdust with new sawdust every 3 days. 

### 4.4. Mouse BMM Preparation and Osteoclastic Differentiation

We obtained bone marrow cells (BMCs) from 5-week-old male ICR mice by flushing tibias and femurs with α-MEM supplemented with 10% FBS, penicillin (100 U/mL), and streptomycin (100 µg/mL). To obtain BMMs, BMCs were cultured in α-MEM supplemented with 10% FBS and M-CSF (10 ng/mL) for 1 day at 37 °C/5% CO_2_ in culture dishes. Nonadherent cells were transferred to 10 cm petri dishes and then cultured in the presence of M-CSF (30 ng/mL) for 3 days. Floating cells were discarded, and cells adhering to the bottom of the culture dish were classified as BMMs. To generate osteoclasts from BMMs, we cultured BMMs in 48-well plates (3.5 × 10^4^ cells/well) at 37 °C/5% CO_2_ for 4 days in the presence of M-CSF (30 ng/mL) and RANKL (100 ng/mL) with different concentrations of methyl gallate (0, 1, 5, 10 μM). After that, the cells were fixed in 3.7% formalin for 15 min, permeabilized with 0.1% Triton X-100 for 10 min, and stained with a TRAP staining solution. TRAP-positive MNCs with more than five nuclei were regarded as osteoclasts.

### 4.5. Evaluation of Cytotoxicity of Methyl Gallate for BMMs

BMMs were seeded in 96-well plates (10^4^ cells/well) in the presence of M-CSF (30 ng/mL) with various concentrations of methyl gallate. After 3 days, 50 µL of tetrazolium salt sodium 3′-{1-[(phenylamino)-carbonyl]-3,4-tetrazolium}-bis (4-methoxy-6-nitro)benzene-sulfonic acid hydrate (XTT reagent) was added into each well followed by incubation for 4 h. The optical density at wavelength of 450 nm was measured by means of a multi-detection microplate reader (Molecular Devices, Sunnyvale, CA, USA).

### 4.6. Quantitative Real-Time Polymerase Chain Reaction (RT-PCR)

Total RNA was isolated using the QIAzol Reagent (Qiagen, Valencia, CA, USA). RNA (1 µg) was reverse-transcribed using oligo-dT primers (10 µg) and dNTPs (10 mM). The mixture was incubated at 65 °C for 5 min and cDNA was obtained by incubation at 42 °C for 50 min with first-strand buffer (50 mM Tris-HCl, pH 8.3; 75 mM KCl; 3 mM MgCl_2_), 100 mM dithiothreitol, RNase inhibitor, and Superscript II reverse transcriptase (Invitrogen, Carlsbad, CA, USA). Real-time RT-PCR was carried out on an Exicycler 96 Real-Time Quantitative Thermal Block (Bioneer Co., Daejeon, Korea) in a 20-µL reaction mixture containing 10 µL of SYBR^®^ Green Premix (Bioneer Co., Daejeon, Korea), 10 pmol each of forward and reverse primer, and 1 µg of cDNA. The amplification conditions were 95 °C for 5 min, followed by 40 cycles of 95 °C for 1 min, 60 °C for 30 s, and 72 °C for 1 min. Fluorescence resulting from the incorporation of the SYBR Green dye into double-stranded DNA was quantified using the threshold cycle (*C*_t_) value. All reactions were run in triplicate, and the data were normalized to glyceraldehyde-3-phosphate dehydrogenase (*GAPDH*). The specific primers used for real-time RT-PCR were as follows: GAPDH: forward, 5′-TCAAGAAGGTGGTGAAGCAG-3′ and reverse, 5′-AGTGGGAGTTGCTGTTGAAGT-3′; c-Fos: forward, 5′-GGTGAAGACCGTGTCAGGAG-3′ and reverse, 5′-TATTCCGTTCCCTTCGGATT-3′; NFATc1: forward, 5′-GAGTACACCTTCCAGCACCTT-3′ and reverse, 5′-TATGATGTCGGGGAAAGAGA-3′; OSCAR: forward, 5′-GGAATGGTCCTCATCTCCTT-3′ and reverse, 5′-TCCAGGCAGTCTCTTCAGTTT-3′; TRAP: forward, 5′-TCATGGGTGGTGCTGCT-3′ and reverse, 5′-GCCCACAGCCACAAATCT-3′; DC-STAMP: forward, 5′-TCCTCCATGAACAAACAGTTCCA-3′ and reverse, 5′-AGACGTGGTTTAGGAATGCAGCTC-3′; OC-STAMP: forward, 5′-ATGAGGACCATCAGGGCAGCCACG-3′ and reverse, 5′-GGAGAAGCTGGGTCAGTAGTTCGT-3′; Atp6v0d2: forward, 5′-GACCCTGTGGCACTTTTTGT-3′ and reverse, 5′-GTGTTTGAGCTTGGGGAGAA-3′; Cathepsin K: forward, 5′-CCAGTGGGAGCTATGGAAGA-3′ and reverse, 5′- CTCCAGGTTATGGGCAGAGA-3′.

### 4.7. Western Blotting

Whole-cell lysates were prepared using lysis buffer consisting of 50 mM Tris-HCl, 150 mM NaCl, 5 mM ethylenediamine tetraacetic acid (EDTA), 1% Triton X-100, 1 mM sodium fluoride, 1 mM sodium vanadate, 1% deoxycholate, and protease inhibitors. The protein content was measured using a Bio-Rad Colorimetric Protein Assay Kit (Bio-Rad Laboratories Inc., Hercules, CA, USA). Equal amounts of protein samples (20 µg by protein) were subjected to SDS-PAGE on 8%–10% gels and were transferred by electroblotting onto polyvinylidene difluoride membranes (Millipore, Bedford, MA, USA). The membranes were blocked with 5% nonfat dry milk and probed for 2 h with the primary antibodies. After washing with tris-buffered saline containing 0.1% Tween 20 (TBST), the membranes were incubated for 1 h with horseradish peroxidase–conjugated sheep anti-mouse IgG antibody or a donkey anti-rabbit immunoglobulin antibody. After a wash with TBST, the specific signals were detected using an Enhanced Chemiluminescence Detection System (Millipore).

### 4.8. Retroviral Gene Transfection

Packaging of the retroviral vectors pMX-IRES-EGFP and pMX-constitutively active (CA) form of Akt-EGFP was performed using transient transfection of these pMX vectors into Plat-E retroviral packaging cells by means of X-tremeGENE 9 (Roche, Nutley, NJ, USA). After incubation in fresh medium for 2 days, the culture supernatants of the retrovirus-producing cells were collected. For retroviral infection, nonadherent BMCs were cultured in M-CSF (30 ng/mL) for 2 days. The BMMs were incubated with a viral supernatant of pMX-IRES-EGFP and pMX-CA-Akt-EGFP virus-producing Plat-E cells together with polybrene (10 µg/mL) and M-CSF (30 ng/mL) for 6 h. The infection efficiency of the retrovirus was determined by green fluorescent protein expression and was always more than 80%. After infection, the BMMs were induced to differentiate into osteoclasts in the presence of M-CSF (30 ng/mL) and RANKL (100 ng/mL) for 4 days. The forced expression of each construct and osteoclast formation were detected using a JuLi™ smart fluorescent cell analyzer (International Medical Products, Brussels, Belgium) and by TRAP staining as described above.

### 4.9. Ca^2+^ Influx Measured by Confocal Microscopy

For detection of the free Ca^2+^ concentration, BMMs were seeded in 12-well plates (4 × 10^4^ cells/well) with incubation for 1 day with M-CSF (10 ng/mL) and were then incubated with M-CSF (30 ng/mL) and RANKL (100 ng/mL) in the presence or absence of methyl gallate (10 µM) for 3 days. The cells were then incubated for 30 min in the presence of 5 µM Fluo-3/acetoxymethyl ester (AM) (Sigma-Aldrich, St. Louis, MO, USA) in serum-free α-MEM. After washing thrice with the same composition of α-MEM, the cells were incubated in α-MEM supplemented with 10% FBS for 30 min, then washed three times with Hank’s balanced salt solution (Gibco BRL, Grand Island, NY, USA) and mounted on slides for confocal microscopy (FluoView FV1000, Olympus, Tokyo, Japan). The cells were excited at 488 nm, and emission was read at 505–530 nm simultaneously at 20-s intervals. To acquire a fluorescent image of intracellular Ca^2+^ concentration and intensity of the Ca^2+^ fluorescent signal stained with Fluo-3/AM, Fluo-3/AM Ca^2+^ indicator-loaded BMMs were analyzed within 80 s.

### 4.10. Filamentous-Actin (F-Actin) Assay

BMMs were incubated with M-CSF (30 ng/mL) and RANKL (100 ng/mL) in the presence or absence methyl gallate (10 µM). After 3 days, the culture medium was replaced with a fresh medium containing the same components. After 1 day, the cells were fixed in 3.7% formalin for 15 min, permeabilized with 0.1% Triton X-100 for 10 min, incubated with 0.25% bovine serum albumin (Sigma-Aldrich, St. Louis, MO, USA) for 30 min, and stained with phalloidin and a 4′,6-diamidino-2-phenylindole (DAPI) solution to visualize F-actin and nuclei, respectively (Life Technologies, Grand Island, NY, USA). Cell fluorescence was detected by means of a laser scanning confocal microscope (Olympus FV1000, Tokyo, Japan). The images were analyzed using the Image-Pro Plus software version 4.0 (Media Cybernetics, Silver Spring, MD, USA).

### 4.11. The Pit Formation Assay

Primary osteoblasts (1 × 10^6^ cells) and BMCs (10^7^ cells) were cultured in collagen gel-coated culture dishes in the presence of 10^−8^ M 1,25-dihydroxy vitamin D_3_ (Sigma-Aldrich) and 10^−6^ M prostaglandin E2 (PGE_2_) (Sigma-Aldrich) for 7 days. The cocultured osteoclasts were detached using 0.1% collagenase at 37 °C for 10 min and were reseeded in 48-well plates, hydroxyapatite-coated plates (Corning, Inc., Corning, NY, USA), or dentin slices in the presence or absence of methyl gallate (10 µM). After 6 h, the cells reseeded in 48-well plates were stained with a TRAP solution. The cells reseeded in hydroxyapatite-coated plates or dentin slices were removed after 24 or 48 h, respectively, and the total number of resorption pits was determined under a microscope and quantified using the Image-Pro Plus software version 4.0 (Media Cybernetics).

### 4.12. The Mouse Model of LPS-Mediated Bone Loss

To determine the effect of methyl gallate in vivo, 5-week-old ICR mice were subdivided into four groups of five mice each and were injected intraperitoneally on days 1 and 4 with LPS (5 mg/kg) or with an equal volume of PBS. Methyl gallate (10 mg/kg) or PBS was administered orally every 8 days. The mice were euthanized after 8 days. Femurs isolated from the mice were washed with PBS and fixed with 4% paraformaldehyde for 1 day. The femurs were analyzed by high-resolution micro-CT (NFR-Polaris-S160; Nanofocus Ray, Iksan, Korea) to obtain 3-dimensional (3D) images (INFINITT-Xelis software; NFINITT Healthcare, Seoul, Korea).

### 4.13. The Micro-Computed Tomography (CT) System

Intact left femur metaphysic regions of each mouse were examined by high-resolution micro-CT (NFR-Polaris-S160; Nanofocus Ray, Iksan, Korea) with a source voltage of 45 kVp, 90 µA current, and 7 µm isotropic resolution. Femur scans were performed over a 2-mm distance from the growth plate, with a total of 350 sections per scan. After 3D reconstruction, bone volume per tissue volume (BV/TV), trabecular separation (Tb.Sp), trabecular thickness (Tb.Th), and trabecular number (Tb.N) were applied to perform quantitative analysis in the INFINITT-Xelis software (INFINITT Healthcare, Seoul, Korea).

### 4.14. Histological Analysis

Femurs were isolated and fixed with 4% paraformaldehyde for 1 day and were decalcified with 12% EDTA (Sigma-Aldrich, St. Louis, MO, USA). The decalcified bones were embedded in paraffin and were cut into slices (thickness of 5 µm) and stained with H&E. Other slices were stained with TRAP to visualize osteoclasts. The number of osteoclasts was determined using the histomorphometric results of the slices stained with TRAP. The number of osteoclasts per visual field of tissue was quantified using the Image Pro-Plus software version 4.0 (Media Cybernetics, Silver Springs, MD, USA).

### 4.15. Statistical Analysis

Experiments were conducted at least three times and the data are expressed as mean ± standard deviation (SD). All statistical analyses were performed by means of the Statistical Package for the Social Sciences Software (SPSS; Korean version 14.0). Student’s *t*-test was performed to compare the parameters between two groups, while the analysis of variance (ANOVA) test followed by the Tukey post hoc test was performed to compare the parameters among three groups. Data with *p* < 0.05 were considered statistically significant.
